# Supplementary material for: Efficient opto- and chemogenetic control in a single molecule driven by FRET-modified bioluminescence
Source: Neurophotonics. 2024 Mar 6;11(2):021005. doi: 10.1117/1.NPh.11.2.021005 (PMC10917299; doi:10.1117/1.NPh.11.2.021005)
Supplement: Supplementary file 1 [file NPh_011_021005_SD001.pdf]

Efficient opto- and chemogenetic control in a single molecule driven by FRET-modified bioluminescence

Andreas Björefeldt, Jeremy Murphy, Emmanuel L Crespo, Gerard G Lambert, Mansi Prakash, Ebenezer C Ikefuama, Nina Friedman, Tariq M Brown, Diane Lipscombe, Christopher I Moore, Ute Hochgeschwender, Nathan C Shaner.

Supplemental Figure S1.

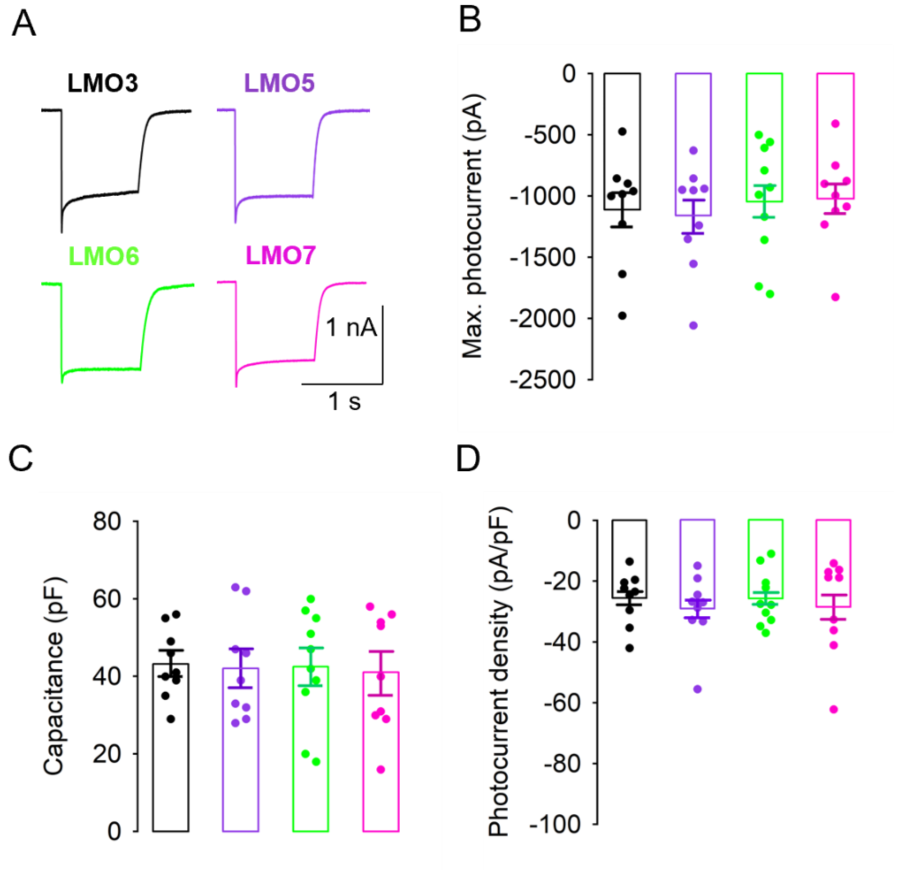

Supplemental Figure S1: Average photocurrent amplitude and density across stable expression HEK lines. (**A**) Example traces of typical photocurrent amplitudes evoked by 1 second illumination at maximum irradiance. (**B**) Summary graph of average VChR1 photocurrent amplitudes evoked at maximum irradiance across HEK lines. (**C**) Summary graph showing capacitance values of all HEK cells and across stable lines. (**D**) Summary graph showing average photocurrent density across HEK lines. Data are presented as mean ± SEM.

Supplemental Figure S2.

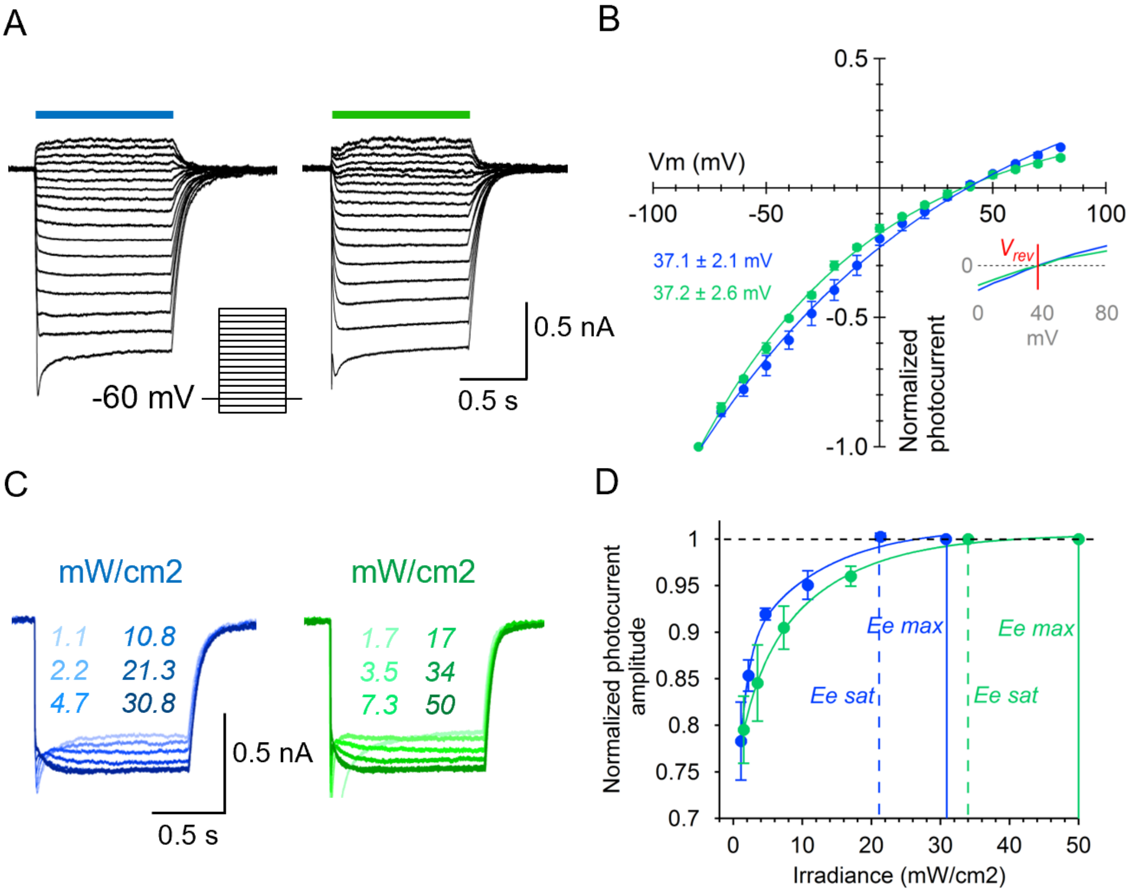

Supplemental Figure S1: Characterization of photocurrent responses to blue and green light in HEK cells. (**A**) Example traces of photocurrent amplitudes evoked by 480 nm (left) and 540 nm (right) light in 1 second windows from -80 to +80 mV. (**B**) Summary graph of photocurrent-voltage relationship and reversal potential of VChR1 with blue and green light illumination (n = 4 cells from LMO3- and LMO7- expressing HEK lines). (**C**) Example traces of photocurrent amplitudes in response to increasing blue (left) and green (right) light intensity as indicated (**D**) Summary graph showing photocurrent amplitude relative to irradiance level with blue and green light (n = 3 LMO3 cells). Ee max; maximum irradiance level obtained, Ee sat; irradiance level sufficient to saturate photocurrent amplitude. Saturation occurs below maximum irradiance levels for both blue and green light. Data are presented as mean ± SEM.

Supplemental Figure S3.

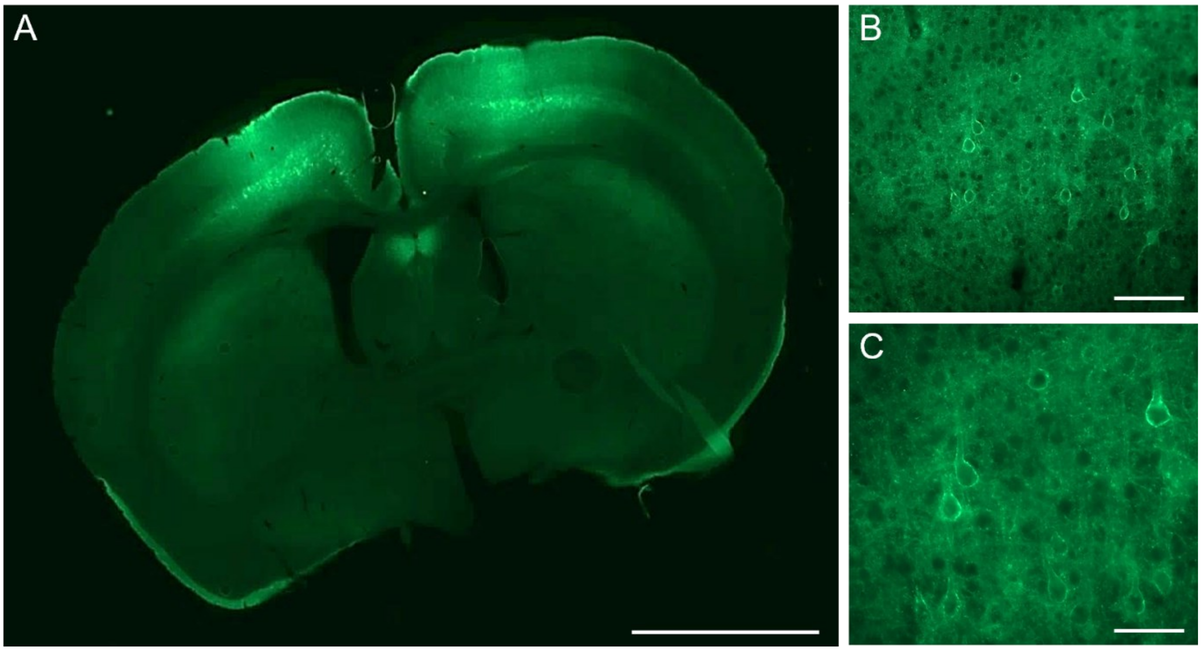

Supplemental Figure S3: Expression of LMO7 in mouse neocortex after early postnatal bilateral AAV injection. (**A**) Representative image showing the distribution of LMO7 (mNeonGreen) fluorescence at 4 weeks post AAV9-hSyn-LMO7 injection into the fronto-paritetal cortex of P2 Swiss Webster mice. (**B**, **C**) Magnified images from ‘A’ showing LMO7-expressing neurons at 20x (**B**) and 40x (**C**). Scale bars; A: 2 mm, B: 200 μm, C: 100 μm.

Supplemental Figure S4.

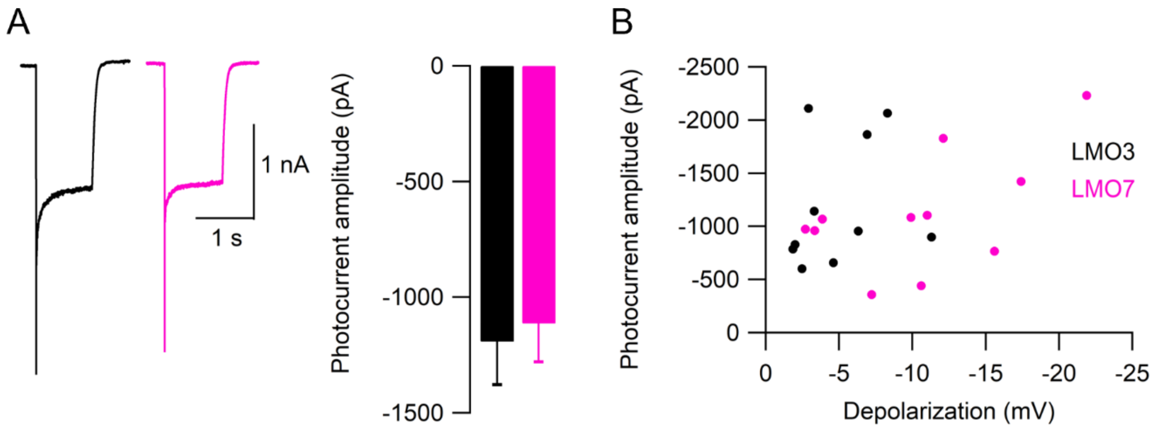

Supplemental Figure S4: Average photocurrent amplitudes recorded from LMO3 and LMO7-expressing neurons. (**A**) Representative photocurrent traces (left) and average photocurrent amplitudes (right) recorded from LMO3 (black) and LMO7 (magenta) populations (480 nm excitation light, 1s). (**B**) Scatter plot showing photocurrent amplitude relative to CTZ-mediated depolarization for each recorded cells (LMO3; n = 10, LMO7; n = 11). Data are presented as mean ± SEM.

Supplemental Figure S5.

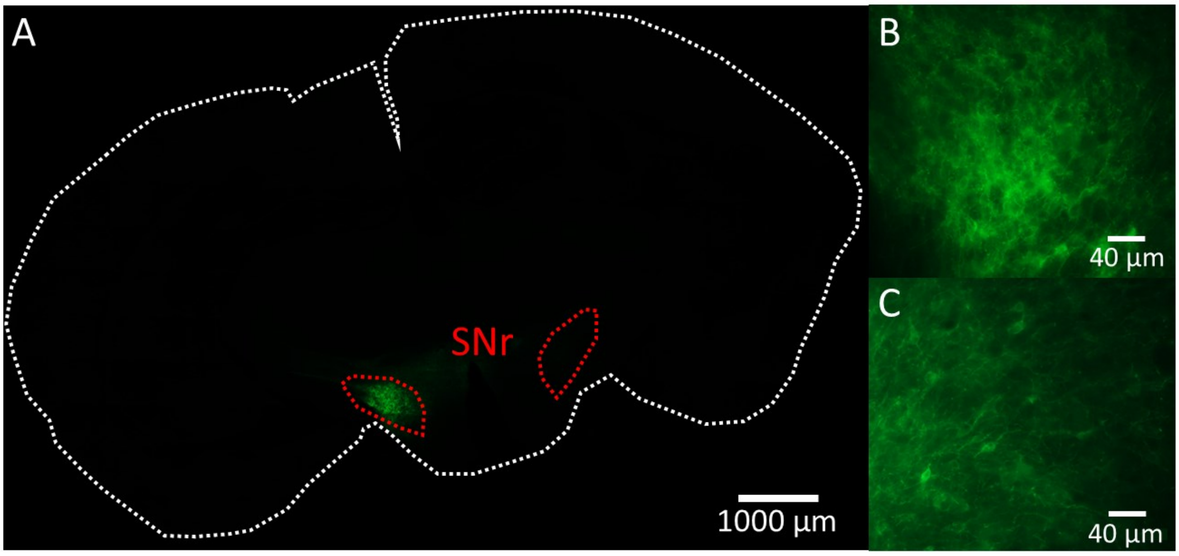

Supplemental Figure S5: Expression of LMO7 in mouse substantia nigra pars reticulata (SNr) after unilateral AAV injection. (A) Representative image showing the localized expression of LMO7 (mNeonGreen) fluorescence at 4 weeks post AAV9-hSyn-LMO7 injection into the left SNr of C57BL/6 adult mice. White stippled line indicates section margins; red stippled lines mark area of SNr on both sites. (B, C) Magnified images from 'A' showing LMO7-expressing neurons at 20x from the center (B) and the periphery (C) of the labeled region.
